# Supplementary material for: Chronic Administration of Non-Constitutive Proteasome Inhibitor Modulates Long-Term Potentiation and Glutamate Signaling-Related Gene Expression in Murine Hippocampus
Source: Int J Mol Sci. 2023 May 3;24(9):8172. doi: 10.3390/ijms24098172 (PMC10179285; doi:10.3390/ijms24098172)
Supplement: Supplementary file 1 [file ijms-24-08172-s001.zip › ijms-2367264-supplementary.pdf]

Supplement

The fEPSP slope represents the tangent of the tilt angle (tg α) for the descending part of the postsynaptic response, corresponding in meaning to the rate of change in postsynaptic potentials. The 100% slope corresponds to the tangent of the tilt angle for the postsynaptic responses before the tetanus or TBS induction in the same slice, whereby an increase in the slope above 100% corresponds to a potentiation caused by one or another electrical stimulation protocol.

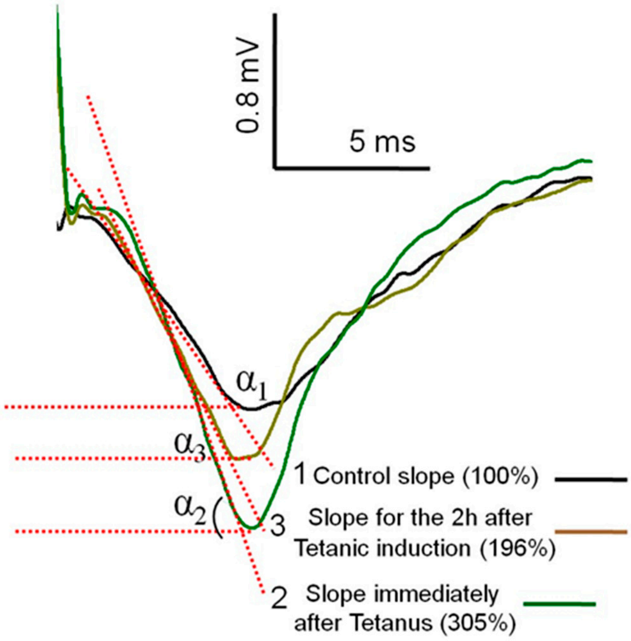

Figure S1. The fEPSP slope estimation in the CA3-CA1 synaptic inputs of hippocampal slices. There are representative recordings for the fEPSP responses in the same slice for: 1 – control recording, before Tetanic induction (black curve), 2 – immediately after Tetanic induction (green curve), and 3 – the 2h after Tetanic induction (dark yellow curve). The fEPSP slope is the tangent of the tilt angle (α1, α2, and α3 for the 1st, 2d, and 3d state of slice throughout experiment, respectively) for the descending part of the postsynaptic response. The average values of the slopes during 20 min (1 measure every 30 s, total 40 sweeps) before the induction of LTP were taken as 100% (control).
